# Phage Display Screening of Bovine Antibodies to Foot-and-Mouth Disease Virus and Their Application in a Competitive ELISA for Serodiagnosis

**DOI:** 10.3390/ijms22094328

**Published:** 2021-04-21

**Authors:** Sukyo Jeong, Hyun Joo Ahn, Kyung Jin Min, Jae Won Byun, Hyun Mi Pyo, Mi Young Park, Bok Kyung Ku, Jinju Nah, Soyoon Ryoo, Sung Hwan Wee, Sang Jick Kim

**Affiliations:** 1 Synthetic Biology and Bioengineering Research Center, Korea Research Institute of Bioscience and Biotechnology, Daejeon 34141, Korea; pearl2926@kribb.re.kr (S.J.); neolub99@naver.com (H.J.A.); 1206rudwls@kribb.re.kr (K.J.M.); 2 Foot-and-Mouth Disease Division, Animal and Plant Quarantine Agency, Gimcheon 39660, Korea; jaewon8911@korea.kr (J.W.B.); hmpyo@korea.kr (H.M.P.); parkmy71@korea.kr (M.Y.P.); kubk@korea.kr (B.K.K.); nahjj75@korea.kr (J.N.); soyooni@korea.kr (S.R.); wsh2010@korea.kr (S.H.W.)

**Keywords:** foot-and-mouth disease virus, type O, type A, phage display, antibody, competitive ELISA

## Abstract

For serodiagnosis of foot-and-mouth disease virus (FMDV), monoclonal antibody (MAb)-based competitive ELISA (cELISA) is commonly used since it allows simple and reproducible detection of antibody response to FMDV. However, the use of mouse-origin MAb as a detection reagent is questionable, as antibody responses to FMDV in mice may differ in epitope structure and preference from those in natural hosts such as cattle and pigs. To take advantage of natural host-derived antibodies, a phage-displayed scFv library was constructed from FMDV-immune cattle and subjected to two separate pannings against inactivated FMDV type O and A. Subsequent ELISA screening revealed high-affinity scFv antibodies specific to a serotype (O or A) as well as those with pan-serotype specificity. When BvO17, an scFv antibody specific to FMDV type O, was tested as a detection reagent in cELISA, it successfully detected FMDV type O antibodies for both serum samples from vaccinated cattle and virus-challenged pigs with even higher sensitivity than a mouse MAb-based commercial FMDV type O antibody detection kit. These results demonstrate the feasibility of using natural host-derived antibodies such as bovine scFv instead of mouse MAb in cELISA for serological detection of antibody response to FMDV in the susceptible animals.

## 1. Introduction

Foot-and-mouth disease virus (FMDV) causes significant foot-and-mouth disease for cloven-hoofed animals [1]. The FMDV is one of the contagious animal disease resulting in social and economic problem due to its rapid spread in countries across the world [2]. The genome of FMDV is a single-stranded RNA (approximately 8.5 kb) that is surrounded by a protein coat consisting of four capsid proteins, namely, VP1, VP2, VP3, and VP4 [3,4]. FMDV occurs in seven major immunologically distinct serotypes: A, O, C, Asia I, and South African Territories (SAT1, SAT2, and SAT3). Of the serotypes, FMDV type O and A have widely spread around the world. Rapid and accurate diagnosis is paramount to limit their distribution and eradiate the diseases.

One of the effective approaches for FMDV diagnosis is to serologically detect FMDV-specific antibodies, which are generated by host immune response against either the viral non-structural proteins (NSPs) or capsid structural proteins (SPs). Unlike NSP antibodies of which presence are used to differentiate infected from vaccinated animals, SP antibodies are detected in both infected and vaccinated animals. Hence, the SP antibody test has been used for FMD surveillance as well as for post-vaccination monitoring. Virus neutralization test (VNT) is a gold standard for detecting SP antibodies, which measures the neutralizing activity of SP antibodies that block virus infection in susceptible cell lines [5]. However, the VNT suffers from such drawbacks as unavoidable use of a live virus, poor reproducibility, and difficulty in large-scale testing. As an alternative, competitive ELISA (cELISA)-based methods have been developed using serotype-specific monoclonal antibodies (MAbs) [6,7]. For example, a commercial kit for FMDV type O antibody includes type O-specific MAb as a key reagent and measures the percent of inhibition (PI) of the MAb binding to antigen by a serum tested. When the PI value is greater than 50%, the serum turns out to be FMDV type O antibody positive. Accordingly, kit performance such as sensitivity and specificity is very dependent on the choice of MAb. The concern about using MAb reagent in cELISA is how well the antibody recognizing a single epitope can represent overall antibody response against diverse epitopes on FMDV capsid. Hence the epitope of antibody reagent in cELISA should be immunodominant and overlap with those of the majority of antibodies produced by an anti-FMDV immune response in host animals.

Diverse sets of MAbs against FMDV have been generated by murine hybridoma technology [7,8,9,10]. Extensive studies on antigenic features of FMDV, especially type O, have identified major neutralizing epitopes recognized by the mouse MAbs [11,12,13]. Even though these epitope regions are suggested to be also recognized by bovine antibodies [14,15], the relative preference of each epitope region and fine epitope structure may be different in cattle. In fact, investigations of antibody response in natural host animals such as cattle, pigs, and sheep have revealed that antibody response does not dominantly direct to the G-H loop of VP1, the historically considered immunodominant epitope of FMDV type O in mice [14,16]. The mouse MAb currently used for cELISA targets the linear epitope on the G-H loop [6] and has such an epitope matching issue that improved performance can be expected from cELISA development using antibody reagent derived from a natural FMDV host animal such as cattle. In addition, the bovine antibody gene repertoire has distinct features when compared with that of a mouse. Mouse germline heavy chain variable (VH) gene segments are diverse in sequence and classified into 16 families, while bovine germline VH gene segments have similar sequences and belong to a single family [17]. Instead, the bovine antibody has extraordinary long heavy-chain complementarity-determining region 3 (HCDR3), which is believed to be the way to achieve high antibody diversity even using the limited source of VH frameworks [18].

Bovine anti-FMDV MAbs can be generated using hetero-hybridoma as well as phage display technology, as reported before [15,19]. Phage display is now considered a better option than hybridoma technology because it erases the instability issue of hybridoma cells, gives more flexibility in antibody selection strategy, and allows higher throughput screening. Recent accumulation of sequence information for bovine germline antibody genes enables the construction of a more reliable antibody library when compared with the first bovine Fab library, which was constructed using limited primer sets [20,21,22]. In this study, we constructed a phage-displayed bovine scFv library from peripheral blood lymphocytes (PBL) of FMDV-vaccinated cattle. Bovine scFv clones with specificity for type O or A as well as pan-serotype could be isolated by panning and screening from the library. Their binding activity and epitope were precisely characterized after the expression and purification of scFv-Fc proteins. Furthermore, the feasibility of their use in cELISA-based serodiagnosis of FMDV was tested using standard FMDV positive sera purchased from Pirbright and serum samples obtained from vaccinated cattle and virus-challenged pigs. Details are reported herein.

## 2. Results

### 2.1. Selection of FMDV-Specific Bovine Antibodies

A bovine scFv library was constructed from PBLs of FMDV-immunized cattle as described in Materials and Methods, and its diversity was estimated 2 × 10^8^ cfu (colony forming unit). To select FMDV-specific antibodies from the library, a library phage was prepared and subjected to panning against both type O (O1 Manisa) and type A (A22 Iraq) of inactivated virus antigens.

After three rounds of panning, the successful enrichment of FMDV binding phage was confirmed by monitoring the phage recovery rate (output/input phage ratio). For both pannings against type O and A, a more than 100-fold increase in the rate was observed after the third round when compared with that after the first round. Most of the scFv phage clones selected randomly after the third round of panning showed FMDV-binding activity in phage ELISA (data not shown), and analysis of their scFv sequences revealed six unique HCDR3 sequences (Table 1). For each HCDR3, a representative scFv phage clone was chosen and tested for its binding activity to type O and A antigens. Three clones with comparable binding activity to both type O and A antigens were named BvOA1, BvOA7, and BvOA18. They could be found from both pannings against type O and A. Two clones (BvO17, BvO22) with type O-specific binding activity were derived from the panning against type O, whereas BvA3, the only clone with preferential binding activity to type A antigen, was selected from the panning against type A.

### 2.2. Characterization of Antibody Specificity

For precise characterization of the selected clones, scFv-Fc expression construct was made for each clone by simple cut-and-paste cloning of scFv insert into the cassette mammalian expression vector pDR-OriP-Fc1 [23]. After transfection of the construct, transient expression in 293E cells resulted in an scFv-Fc molecule of 55 kDa secreted as dimers of 110 kDa, which was purified from the culture supernatant by affinity chromatography on a protein G column. The purity and integrity of the purified scFv-Fcs were confirmed by SDS-PAGE analysis, which showed more than 95% purity of scFv-Fcs (Figure S1).

**Figure 1:**
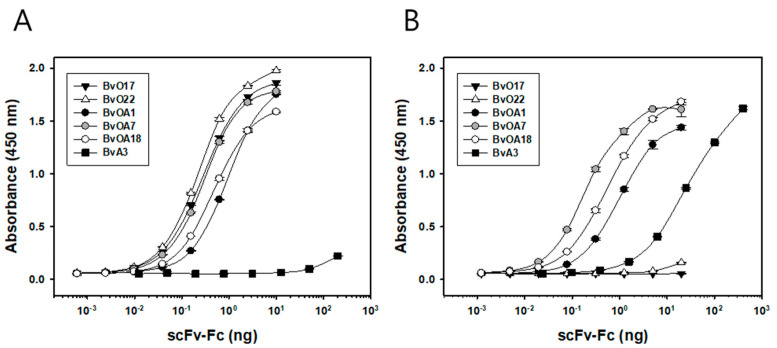
Binding activities of selected bovine scFvs toward (**A**) FMDV type O (O1 Manisa) and (**B**) FMDV type A (A22 Iraq) antigens. scFv sequences selected by phage library screening of FMDV-immunized cattle were cloned into a pDR-OriP-Fc1 vector, which allowed scFv-Fc expression in mammalian cells. Serial dilutions of purified scFv-Fc were applied onto FMDV antigen-coated 96-well Maxisorp plate. Bound scFv-Fc proteins were detected using HRP-conjugated anti-human IgG (Fc-specific). Data are shown as mean ± SD of triplicate samples.

The binding affinity of the purified scFv-Fcs for either type O or A antigen was measured by an indirect ELISA (Figure 1). As in Table 2, the apparent dissociation constant (K_D_) for each binding interaction was determined by binding curve fitting as described previously [24]. As expected, BvOA1, BvOA7, and BvOA18 bound to both O1 Manisa and A22 Iraq antigens in a concentration-dependent manner. Their affinities appeared to be very high for both strains of FMDV, with K_D_ values ranging from 18.5 to 93.3 pM. BvO17 and BvO22 also showed very high affinity for O1 Manisa with K_D_ values of about 20 pM but negligible binding activity for A22 Iraq. BvA3 showed preferential binding to A22 Iraq, and its affinity was relatively low when compared with those of the other clones.

**Figure 2:**
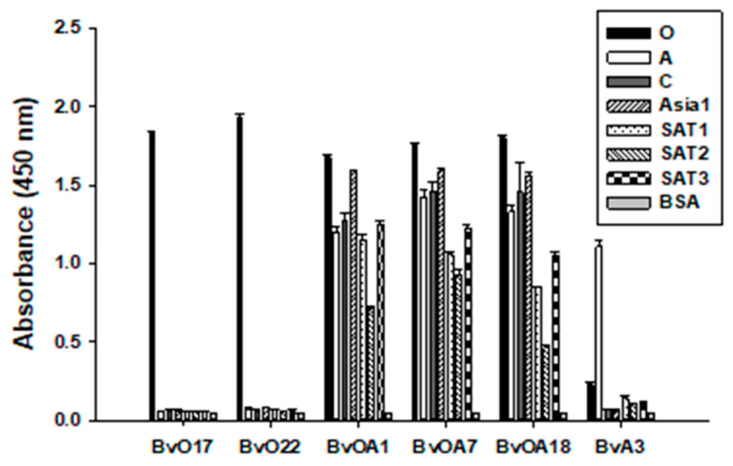
Cross-reactivities of bovine scFvs to seven serotypes of FMDV. Inactivated FMDV antigens for seven serotypes purchased from Pirbright Institute and BSA (negative control) were coated onto a 96-well Maxisorp plate. A total of 0.1 µg/mL of purified scFv-Fc was applied onto the wells for most clones except BvA3, of which 2 µg/mL was applied. Bound scFv-Fc proteins were detected using HRP-conjugated anti-human IgG (Fc-specific). Data are shown as mean ± SD of triplicate samples.

Next, we further tested the cross-reactivity of scFv-Fcs for the other serotypes (Figure 2). As expected, BvOA1, BvOA7, and BvOA18 were reactive to all the tested serotypes of FMDV serotypes. They may recognize common epitopes of FMDV regardless of serotype and can be used for pan-serotype diagnosis of FMDV. As clearly shown in Figure 2, BvO17 and BvO22 specifically bound to O1 Manisa and were not cross-reactive with the other serotypes of FMDV strains tested. It is likely that they recognize type O-specific epitope and can be used for the detection of type O-specific antibody response. Unlike the type O-specific clones, BvA3 was not perfectly specific to type A. Even though BvA3 bound to A22 Iraq preferentially, it seemed to be still reactive to other serotypes of strains, especially O1 Manisa.

### 2.3. Characterization of the Antibody Binding Epitopes

**Figure 3:**
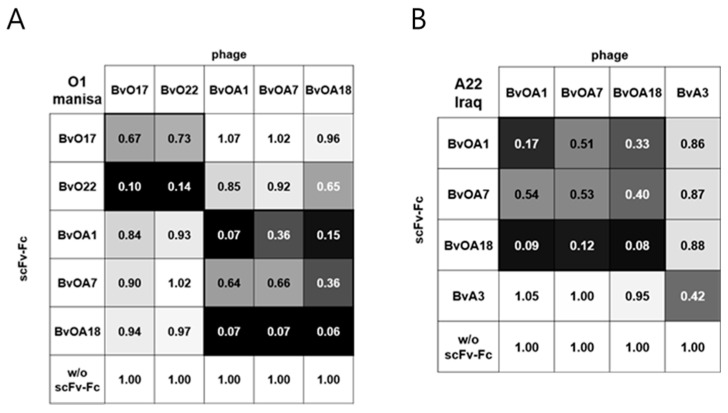
Epitope binning of bovine scFvs by cross-competition ELISA. Inactivated FMDV type O (O1 Manisa) (**A**) or type A (A22 Iraq) (**B**) antigens were coated onto a 96-well Maxisorp plate. A total of 10 µg/mL of purified scFv-Fc was added as a competitor, followed by incubation with scFv phage. Bound scFv phages were detected using HRP-conjugated anti-M13 antibody. The data represents normalized values obtained by dividing the absorbance values by that of scFv phage binding in the absence of competitor scFv-Fc. Strong binding inhibition is also represented by the darker background in the cell.

To investigate whether the selected clones recognize overlapping epitopes or not, cross-competition binding experiments for both O1 Manisa and A22 Iraq antigens were carried out using scFv phage and scFv-Fcs. Figure 3 shows antibody epitope binning data, and the value in each cell represents relative absorbance for the binding of scFv phage shown on the upper side in the presence of competitor scFv-Fc shown on the left side. The degree of binding inhibition was also expressed as the darkness of the cell background, which allowed easy grouping of antibodies according to their epitopes. Pan-serotype antibodies, BvOA1, BvOA7, and BvOA18, competed with each other for binding to both types of antigens suggesting their recognition of overlapping epitopes common to all serotypes. Type O-specific antibodies, BvO17 and BvO22, also competed with each other for binding to type O antigen, which indicated a shared epitope region on type O antigen recognized by both. Since BvA3 did not compete with the other antibodies, three different groups could be finally defined in terms of epitope specificity.

### 2.4. Detection of FMDV Antibody in Serum by cELISA Using Bovine Antibodies

To test whether the selected bovine antibodies can be used for the detection of serum antibodies against SP of FMDV, a solid-phase competitive ELISA (SPCE) was developed using biotinylated scFv-Fcs as described in Materials and Methods. In the SPCE, binding of biotinylated scFv-Fcs to FMDV antigen is detected by premixed to NeutrAvidin-HRP (NA-HRP) and can be inhibited by the presence of anti-SP antibodies in test sera. Because BvA3 lost its binding activity after biotinylation, intact scFv-Fcs was instead used together with anti-human IgG (Fc-specific)-HRP. After a preliminary test using a small size of serum samples from vaccinated cattle and virus-challenged pigs (Figure S2), BvO17 and BvOA7 were selected as representative clones for the respective groups since they showed slightly better response than the other clones in the same group. The final three clones, BvO17, BvA3, and BvOA7, were further tested for the SPCE to detect antibodies against FMDV type O and A. PrioCHECK Kits for type O and A were used for comparison in this study since PrioCHECK family of type-specific FMDV antibody tests are widely used as commercial SPCE kits for primary screening.

First, strong positive control sera for each serotype of FMDV and a negative control serum purchased from Pirbright Institute were tested in SPCE (Figure S3). When BvO17 was used as a reagent, its binding was significantly reduced by type O serum (Figure S3B). Except for type Asia1 serum, it showed very weak or negligible cross-reactivity for non-type O control sera. In all the tested SPCE, type Asia1 serum was reactive regardless of serotype specificity of antibody reagent used. The pattern of binding inhibition by the control sera in BvO17-based SPCE was very similar to that in PrioCHECK type O Kit (Figure S3A,B). BvA3-based SPCE showed significant binding inhibition by type A serum and weak or negligible cross-reactivity for non-type A control sera except type Asia1 serum (Figure S3E). Unlike BvA3-based SPCE, PrioCHECK type A Kit did not show comparable binding inhibition by type A serum (Figure S3D,E). When excluding the cross-reactive type Asia1 serum, these results indicate BvO17 and BvA3 can be used for serotype-specific detection of FMDV antibodies. In the case of the pan-serotype antibody, BvOA7, its binding inhibition was observed for most of the sera tested. However, the degree of inhibition was dependent on the serotype as well as the FMDV antigen used (Figure S3C,F).

**Figure 4:**
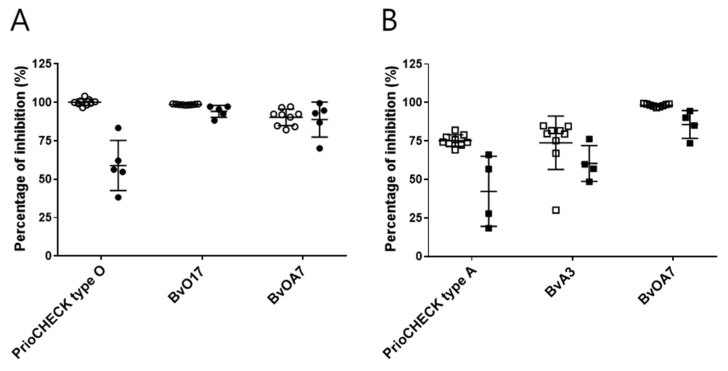
Detection of FMDV antibodies in the serum samples from vaccinated and virus-challenged animals using bovine scFv antibody-based SPCE. (**A**) For type O antibody detection in the serum samples from vaccinated cattle (n = 9, ○) and FMDV (O/Anseong/SKR/2002)-challenged pigs (n = 5, ●), BvO17 and BvOA7-based SPCEs were carried out as described in Figure S3, and their performance was compared with a parallel experiment using PrioCHECK type O antibody ELISA Kit. The results were expressed as a percentage of inhibition as described in Materials and Methods. (**B**) For type A antibody detection in the serum samples from vaccinated cattle (n = 9, □) and FMDV (A/Yeoncheon/SKR/2017)-challenged pigs (n = 4, ■), BvA3 and BvOA7-based SPCEs were carried out as described in Figure S3, and their performance was compared with a parallel experiment using PrioCHECK type A antibody ELISA Kit.

Next, serum samples from vaccinated cattle and virus-challenged pigs were tested in SPCE. The bovine sera were collected from a local slaughterhouse and were supposed to be both positive for type O and A FMDV antibodies due to national vaccination programs in South Korea. The porcine sera expected to be positive for FMDV type O or A antibodies were collected from pigs exposed to infection with Korean isolates, either FMDV (O/Anseong/SKR/2002) or FMDV (A/Yeoncheon/SKR/2017). In BvO17-based SPCE (Figure 4A), the bovine sera were strongly positive, exhibiting PI values near 100%. The values were comparable with those obtained by PrioCHECK type O Kit. The porcine sera derived from FMDV (O/Anseong/SKR/2002)-challenged pigs provided relatively lower PI values than the bovine sera but still had a high average PI value, 94%. When compared with the PrioCHECK type O Kit, which showed a low average PI value (only 59%) with large variance, BvO17-based SPCE seemed to be much better in the sensitive detection of the porcine FMDV antibodies. In BvA3-based SPCE (Figure 4B), the average PI value was 74% for bovine sera, which was comparable with the PrioCHECK type A Kit. However, the variance was huge, unlike the PrioCHECK type A Kit, and even one sample showed a PI value far below 50%. The PI values for the porcine sera derived from FMDV (A/Yeoncheon/SKR/2017)-challenged pigs were relatively low with an average of 60% and showed wide fluctuations. PrioCHECK type A Kit provided even worse data for the porcine sera exhibiting the average PI value less than 50%, also with huge variance. In the case of BvOA7-based SPCE (Figure 4A,B), the PI values were sufficiently high for both type O and A formats, indicating that they can be generally used for detection of the FMDV antibody, regardless of serotype.

## 3. Discussion

In this study, we successfully isolated bovine anti-FMDV antibodies by phage display using scFv library generated from PBLs of cattle in a local slaughterhouse. The cattle were considered to be adequate for the FMDV-immune antibody library construction because they were supposed to be immune to both FMDV type O and A due to a vaccine policy in South Korea. In fact, they were seropositive for FMDV type O and A when checked with PrioCHECK FMDV antibody detection kits. The bovine scFv library constructed was hence presumed to contain antibodies against FMDV type O and A. Through two separate pannings against type O and A antigens, we could obtain serotype (O or A)-specific scFvs that distinguish other serotypes. In addition, scFv clones reactive to both type O and A antigens could be isolated and further characterized to be cross-reactive to all seven serotypes.

It is well known that the bovine antibody repertoire is derived from a highly limited diversity of germline immunoglobulin variable region genes [17,20,21,25]. For germline VH genes, there are 12 functional VH gene segments, all of which belong to one subgroup, VH1. For germline VL genes, the Vλ1 subfamily dominantly participated in the bovine light chain repertoire that consists of 95% of lambda (λ) and 5% of kappa (κ) light chains expressed. The sequence analysis of our bovine scFvs revealed VH1 and Vλ1 as the only germline VH and VL gene subfamily, respectively (Table 1). For the six selected bovine scFvs, the germline VH1-10 gene segment was most frequently used in our study. It was also one of the two most frequently used germline VH genes in the recent report on analysis of germline gene use of 55 plasmablast-derived MAbs from FMDV-infected cattle [26]. This high frequency of VH1-10 can be simply attributed to its abundance in bovine antibody repertoire rather than FMDV-specific selection. In fact, VH1-10 has been found in about 70% of sequences in a recent analysis of deep sequencing of bovine VH repertoire [27]

Another feature in the bovine antibody sequence is the long length of HCDR3, which is believed to compensate for the limited diversity of V-D-J recombination. The average length is well over 20 a.a. residues and about 10% of the antibodies have extremely long HCDR3 of between 40 and 70 a.a. [18,28,29]. This ultra-long HCDR3 could be also identified by sequence analysis of the plasmablast-derived anti-FMDV MAbs in the previous study. However, all the selected HCDR3s in our study were normal (from 14 to 29 a.a.) in length. Our scFvs went through repetitive panning rounds, which might enrich binders that have advantages in affinity and expression. Hence, we can assume that they may have such advantages over antibodies with ultra-long HCDR3 and become final survivors after in vitro selection procedure.

Studies on the antigenic profile of FMDV type O using mouse MAbs have revealed five neutralizing epitope sites [11,12,13]. Site 1 is well known as an immunodominant and linear epitope that includes the G-H loop in VP1, whereas the other sites are all conformational epitopes [30,31]. When the bovine MAbs developed by heterohybridoma technology were tested for epitope specificity [15], none of them recognized site 1. Instead, they all recognized the other conformational epitope sites. Similarly, most bovine plasmablast-derived MAbs were found to be specific to conformational epitopes outside of site 1 [26]. Thus, unlike in mice, immunodominant epitopes for FMDV type O in cattle seemed to be conformational rather than linear. BvO17 and BvO22 were selected as type O-specific clones with high affinity in this study. Despite no sequence homology in the HCDR3 region, they showed similar epitope specificity in cross-competition ELISA (Figure 3). When they were tested for western blotting of capsid protein, none could succeed (data not shown). This result indicates that they recognize conformational epitopes but not site 1 at least and may reflect the previous suggestion that antibody response against FMDV type O in cattle differs from that in mice and does not interact dominantly with the linear epitope in the G-H loop of VP1. In this study, we did not further characterize the epitopes recognized by the bovine scFv antibodies. Since they are conformational, it may be difficult to fully characterize the epitope structure. The clues for the structure can be given by several experimental tools such as binding test with recombinant viral capsid proteins, viral epitope library scan, and selection and identification of the antibody-resistant mutant virus. Such endeavors are very important and will contribute to the understanding of the performance of bovine scFv in cELISA and the further development of improved antibody reagent.

This difference in epitope specificity affected the performance of SPCE for the detection of FMDV type O antibodies. PrioCHECK kit includes HRP-labeled mouse MAb as a key reagent. Like the previous report on solid-phase blocking ELISA for detection of FMDV type O antibody [6], the MAb also targeted the linear epitope in the G-H loop when we checked specificity using synthetic peptide (Data not shown). Compared with the mouse MAb-based PrioCHECK kit, BvO17-based SPCE showed a similar competition pattern for controlling bovine anti-FMDV sera for seven serotypes (Figure S3). However, it was better in the sensitive detection of FMDV type O antibody, especially for serum samples from virus-challenged pigs (Figure 4). This result may indicate that more antibodies are developed against the conformational epitope region recognized by BvO17 than the linear epitope in the G-H loop when cattle and pigs are exposed to FMDV serotype O. It seems to be promising to develop BvO17-based SPCE for sensitive detection of FMDV type O antibody in both bovine and porcine serum samples.

Unlike BvO17, BvA3 did not provide good performance in SPCE. Though BvA3-based SPCE showed better serotype A-specific competition than the PrioCHECK type A kit in a test using control bovine anti-FMDV sera for seven serotypes, its sensitivity turned out to be low in tests using the serum samples from vaccinated cattle and virus-challenged pigs. PrioCHECK type A kit also showed low sensitivity, especially for the porcine samples. More sensitive detection of FMDV type A antibody in serum samples may require further screening of natural host-derived antibodies recognizing immunodominant epitope that is unique to type A. For such purpose, screening of antibody repertoire from host animals immunized with type A only can be considered as an alternative option since it is reasonable to speculate that our approach using both type O- and A-vaccinated cattle provided rare type A-specific antibody clones due to biased enrichment of antibody clones recognizing both serotypes during in vitro selection procedure.

BvOA7-based SPCE could be used successfully to detect both FMDV type O and A antibodies. When it comes to the detection of type A antibodies, it showed even better sensitivity than the BvA3-based SPCE and PrioCHECK type A kit (Figure 4B). Due to pan-serotype specificity, BvOA7 cannot be used to differentiate serotypes but can be developed as a general detection reagent for FMDV antibodies regardless of serotypes. Other than type-specific assay, there is also a need for a universal serological test as simplified frontline diagnostics as described recently [32].

It looks like some data in Figure S3 seem to be inconsistent with the data in Figure 4. For example, BvOA7-based SPCE and PrioCHECK type A kit did not respond effectively to type A reference serum sample (Figure S3) while both responded very well to vaccinated bovine serum samples (Figure 4B). Since Figure S3 is derived from a single serum sample for each serotype, it has a limitation of data interpretation even though each sample is purchased as a reference control serum for each subtype. When considering sample to sample variation, one should not interpret the data in Figure S3 as a representation for a specific serotype. Each bar in Figure S3 can be just one of the dots in the plot of Figure 4. Hence we relied on Figure 4, representing data collection for diverse test serum samples, to judge the feasibility of using our antibodies in cELISA. There may still remain concerns about a limited number of serum samples tested. As this was a preliminary feasibility study for the bovine scFvs selected, the serum samples were not broadly representative, and the sample size was not big enough. For further validation study, we will expand sample size and diversity by adding positive and negative samples from a broad range of host animals and virus isolates, which can meet the requirement for commercial development.

In conclusion, we herein demonstrate the isolation of anti-FMDV bovine scFv clones specific to a serotype (O or A) as well as those with pan-serotype specificity by phage library screening of antibody repertoire from FMDV-vaccinated cattle. Their performance in SPCE showing better sensitivity than conventional mouse MAbs suggests the feasibility of their application in serodiagnosis of FMDV. The benefit of using the bovine scFvs for FMDV serodiagnosis will be further proved by a validation study using a large number of diverse field serum samples, including negative serum samples. In parallel, further engineering of them as antibody reagents can also be considered for optimization of performance through direct genetic fusion with detection modules such as HRP and fluorescent proteins. Such endeavors will allow commercial development of a novel bovine scFv-based cELISA as a more reliable tool for FMDV surveillance and ultimately contribute to control FMDV spread in the world.

## 4. Materials and Methods

### 4.1. Construction of Bovine scFv Library

Blood samples of ten Hanwoo (*Bos Taurus coreanae*) cows were collected from a local slaughterhouse. Peripheral blood lymphocytes (PBLs) were isolated using Histopaque-1077 (Sigma-Aldrich, St. Louis, MO, USA) and subjected to total RNA extraction with Trizol (Thermo Fisher Scientific, Waltham, MA, USA) to obtain a high percentage of inhibition (PI) values when tested by PrioCHECK FMDV type O Antibody ELISA Kit (Thermo Fisher Scientific, Waltham, MA, USA). cDNA was synthesized using Superscript IV (Thermo Fisher Scientific, Waltham, MA, USA) and used for PCR amplification of bovine antibody variable regions, VH and VL (Vκ and Vλ). The PCR primer set used for each variable region (Table S1) was designed by considering the sequences of bovine germline antibody gene segments as described previously [33]. The amplified VH and VL sequences were combined by assembly and extension PCR using extension primers (Table S1), and the resulting scFv sequences with (G_4_S)_3_ linker between VH and VL were cloned into phagemid vector, pDR-D1 as described before [23]. Bacteriophages displaying the scFv repertoire were prepared from the transformed *E.coli* ER2738 cells using VCSM13 helper phage (Stratagen, La Jolla, CA, USA) as described previously [23].

### 4.2. Bio-Panning and Screening

Immunotubes (Nunc, Maxisorp, Thermo Fisher Scientific, Waltham, MA, USA) were coated with either inactivated FMDV type O (O1 Manisa) or type A (A22 Iraq) antigen (Pirbright Institute, Pirbright, U.K.) for panning. The tubes were washed twice with PBS and blocked with 2% skim milk in 1X PBS supplemented with final 0.05% Tween-20 (PBST). The library phages were added to the antigen-coated tube and incubated at room temperature for 2 h. Then, unbound phages were removed by PBST washing five times. A total of 0.2 M Glycine-HCl (pH 2.7) was used to elute bound phage, and 1 M Tris-HCl (pH 8.0) was added for neutralization. The eluted phages were infected with *E.coli* ER2738 cells, followed by their superinfection with VCSM13 helper phage for amplification. The amplified phages were used for the next round of panning with an increased number of times of PBST washing.

To screen individual scFv phage clones, forty-eight colonies were randomly selected from the output plates after the third round of panning for each panning procedure. After growing them until OD_600_ = 0.5, scFv phages were rescued by superinfection with helper phage. The rescued phages were applied onto the FMDV-coated 96-well Maxisorp plate (Nunc, Maxisorp). The phage binding was detected by phage ELISA using horseradish peroxidase (HRP)-conjugated anti-M13 antibody (Sino Biological, Beijing, China). The clones showing specific binding activity were subjected to DNA sequencing, and the nucleotide sequences determined were submitted to IMGT/V-QUEST (http://imgt.org/IMGT_vquest/input, last accessed December 2, 2020) for sequence analysis.

### 4.3. Expression and Purification of scFv-Fc Proteins in 293E Cells

The genes encoding the selected scFv clones were cloned into the pDR-OriP-Fc vector, which allows genetic fusion of scFv sequences to human gamma-1 Fc and hinges domain sequences for transient expression of scFv-Fc as described previously [23]. The constructed expression plasmid was transfected using polyethyleneimine (PEI, Polysciences, Warrington, PA, USA) into 293E (CRL-10852, ATCC) cells cultivated in suspension with Ex-Cell 293 serum-free medium (Sigma-Aldrich, St. Louis, MO, USA) at 37 ℃ in 8% CO_2_. The transfected cells were cultured for seven days at 32 ℃ in 8% CO_2_ while being fed with 15% glucose (Thermo Fisher Scientific, Waltham, MA, USA) and 200× Glutamax (Thermo Fisher Scientific, Waltham, MA, USA) twice. For purification of scFv-Fc, the supernatant was subjected to affinity chromatography based on a protein G-agarose column (Merck Millipore, Darmstadt, Germany).

### 4.4. ELISA

96-well Maxisorp plate (Nunc, Maxisorp, Thermo Fisher Scientific, Waltham, MA, USA) were coated with FMDV antigens (O1 Manisa or A22 Iraq) (Pirbright Institute, Pirbright, U.K.) diluted in PBS (pH 7.4) overnight at 4 °C, and blocked with 2% skim milk or 1% BSA in PBST. The plates were washed four times with PBST between steps. All incubations were carried out at RT for 1-2 h. The color was developed with tetramethylbenzidine (TMB) substrate reagents (BD Biosciences, San Diego, CA, USA), and the reaction was stopped with 50 µL of 2.5 M H_2_SO_4_. The absorbance was measured at 450 nm (A_450_) using a microtiter reader (Emax, Molecular Devices, Sunnyvale, CA, USA).

The binding activities of the purified scFv-Fc were measured by an indirect ELISA. Serial dilutions of scFv-Fc were applied to FMDV antigen-coated wells and bound scFv-Fc was detected by HRP-conjugated anti-human IgG antibody produced in goat (Jackson ImmunoResearch, West Grove, PA, USA). From the binding data, equilibrium dissociation constant, K_D_ was estimated as described previously [24]. By plotting absorbance as y and antibody concentration applied as x, K_D_ can be calculated by nonlinear regression using hyperbola model equation, y = A_max_ ∗ x/(K_D_ + x).

For antibody cross-competition binding experiment, 10 µg/mL of purified scFv-Fc was added as a competitor into each well and incubated for 1 h at RT. After washing, an scFv phage diluted with 2% skim milk in PBST was added into each well and incubated for 45 min at RT. After washing, HRP-conjugated anti-M13 monoclonal antibody (1:5000 dilution) was used to detect bound phage.

### 4.5. Biotinylation of scFv-Fc

The scFv-Fc proteins were conjugated with Biotin using a Sulfo-NHS-LC-Biotin (sulfosuccinimidyl-6-(biotinamido) hexanoate; Thermo Fisher Scientific, Waltham, MA, USA) according to the manufacturer’s instructions. A total of 10 mM of Sulfo-NHS-LC-Biotin reagent was added to 1 mg of scFv-Fc proteins and incubated at 4 °C for 2 h. The mixture was then washed using amicon ultra centrifugal tube (10 K) several times and stored at 4 °C until further use.

### 4.6. Serum Samples

As reference sera, bovine control sera strongly positive for seven serotypes (O/UKG, A/A22, C/Oberbayern, SAT1/RHO, SAT2, SAT3/ZIM, and Asia1/Shamir) as well as negative control serum were purchased from Pirbright Institute. Bovine test sera were prepared from blood samples collected from cattle at a local slaughterhouse that were supposed to be immunized by both FMDV type O and A vaccines. Porcine test sera were collected from pigs challenged either by FMDV (O/Anseong/SKR/2002) or by FMDV (A/Yeoncheon/SKR/2017) with support from the Animal and Plant Quarantine Agency (Gimcheon, South Korea).

### 4.7. Solid-Phase Competitive ELISA (SPCE)

The biotinylated scFv-Fc together with NeutrAvidin-HRP (NA-HRP, Thermo Fisher Scientific, Waltham, MA, USA) was used to develop bovine scFv-based SPCE. In the case of BvA3, unlabeled scFv-Fc together with HRP-conjugated anti-human IgG (Fc-specific) was used since it lost binding activity after biotinylation. For detection of FMDV type O or A antibodies, a 96-well Maxisorp plate was coated overnight at 4 ℃ with FMDV type O (O1 Manisa) or type A (A22 Iraq) antigens, respectively. The wells were blocked with 1% BSA in PBST at RT for 1 h. A total of 1:10 dilutions of serum samples were added to the wells and incubated at RT for 1 h. After washing with PBST, either a premix of biotinylated scFv-Fcs and NA-HRP or a premix of unlabeled scFv-Fc and HRP-conjugated anti-human IgG (Fc-specific) was added and incubated for 45 min at RT. The wells were washed four times with PBST, followed by incubation with 100 µL of TMB substrate for 10 min and subsequent addition of 2.5 M H_2_SO_4_. A_450_ was measured for each serum sample. Percentage of inhibition (PI) was calculated using the following formula Equation (1):

(1) $$\mathrm{PI}\left(\%\right)=\left\{1-\left(\frac{A_{450}test\;serum}{A_{450}negative\;reference\;serum}\right)\right\}\times 100$$

For comparison, commercial SPCE (PrioCHECK FMDV type O or A antibody ELISA Kit; Prionics AG, Schlieren-Zurich, Switzerland) was also carried out in parallel according to the manufacturer’s instructions. Briefly, 1:10 (type O kit) or 1:5 (type A kit) dilution of serum was incubated in the respective FMDV antigen-coated wells. After washing, dilution of HRP-conjugate provided by the kit was added to the wells and incubated for 1 h at RT. After removing unbound conjugate by repeated washes, color was developed by adding 100 µL of TMB substrate solution and incubating for 15 min. Then the reaction was stopped by adding 100 µL of stop solution. PI value was calculated according to the manufacturer’s instructions.

## Figures and Tables

**Table 1:** Analysis of VH and VL gene usage in the selected scFv clones. The nucleotide sequences were analyzed using IMGT/V-QUEST (http://www.imgt.org/IMGT_vquest/input, last accessed December 2, 2020).

Clone	Bovine Germline VH Segment	Differences from Germline (nt, aa)	HCDR3 (IMGT)	Bovine Germline VL Segment	Differences from Germline (nt, aa)	LCDR3 (IMGT)
BvO17	IGHV1-17*01	(21, 13)	AKCSHEYANYACYDFEDESYFDA	IGLV1-47*01	(14, 9)	AAHDSSINNGV
BvO22	IGHV1-10*01	(17, 9)	AKEADDDADHCADLDI	IGLV1-67*02	(16, 8)	VTYDSTSSTDL
BvOA1	IGHV1-10*01	(20, 8)	AKYAGDHGISGDGCYAFAVGYVDA	IGLV1-67*02	(40, 22)	VVYDSAKDTAI
BvOA7	IGHV1-10*01	(22, 12)	AKNMGDMGSCYAWANGYVDA	IGLV1-67*02	(25, 13)	AAYDSSSNAV
BvOA18	IGHV1-21*01	(16, 7)	AKGYDAGYTADCIYDYGYGRERYVDA	IGLV1-47*01	(16, 9)	ASPGGSSTNAV
BvA3	IGHV1-10*01 F or IGHV1-10*02 F	(15, 8)	TKDVNLYNLWSGGGYGCFGDGRGIDYNYVDT	IGLV1-67*01 F	(24, 11)	ATGDYSSSTSI

**Table 2:** Binding affinity of selected scFv clones. Asterisks (*) are not detected.

	Binding Affinity (K_D_, pM)	
	O1 Manisa	A22 Iraq
BvO17	25.9 ± 1.9	* N.D.
BvO22	21.8 ± 1.4	* N.D.
BvOA1	93.3 ± 9.0	91.8 ± 9.0
BvOA7	27.9 ± 1.2	18.5 ± 1.3
BvOA18	46.1 ± 3.9	50.5 ± 5.3
BvA3	* N.D.	2179.9 ± 322.0

## Supplementary Materials

The following are available online at https://www.mdpi.com/article/10.3390/ijms22094328/s1.
