# Supplementary material for: Phage Display Screening of Bovine Antibodies to Foot-and-Mouth Disease Virus and Their Application in a Competitive ELISA for Serodiagnosis
Source: Int J Mol Sci. 2021 Apr 21;22(9):4328. doi: 10.3390/ijms22094328 (PMC8122579; doi:10.3390/ijms22094328)
Supplement: Supplementary file 1 [file ijms-22-04328-s001.zip › ijms-1158520-supplementary.pdf]

Supplementary Materials

Phage Display Screening of Bovine Antibodies to Foot-and-Mouth Disease Virus and Their Application in a Competitive ELISA for Serodiagnosis

Sukyo Jeong[1], Hyun Joo Ahn[1], Kyung-Jin Min[1], Jae-Won Byun[2], Hyun Mi Pyo[2], Mi-Young Park[2], Bok Kyung Ku[2], Jinju Nah[2], Soyoon Ryoo[2], Sung-Hwan Wee[2] and Sang Jick Kim[1, *].

[1] Synthetic Biology and Bioengineering Research Center, Korea Research Institute of Bioscience and Biotechnology, Daejeon 34141, Korea

[2] Foot-and-Mouth Disease Division, Animal and Plant Quarantine Agency, Gyongsangbuk-do 39660, Korea

Figure S1. Expression and purification of scFv-Fcs using HEK293E cells. 12% SDS-PAGE analysis of the purified scFv-Fcs by affinity chromatography using Protein G-agarose resin. All the scFv-Fcs were detected at the predicted molecular weight of around 55 kDa (black arrow).

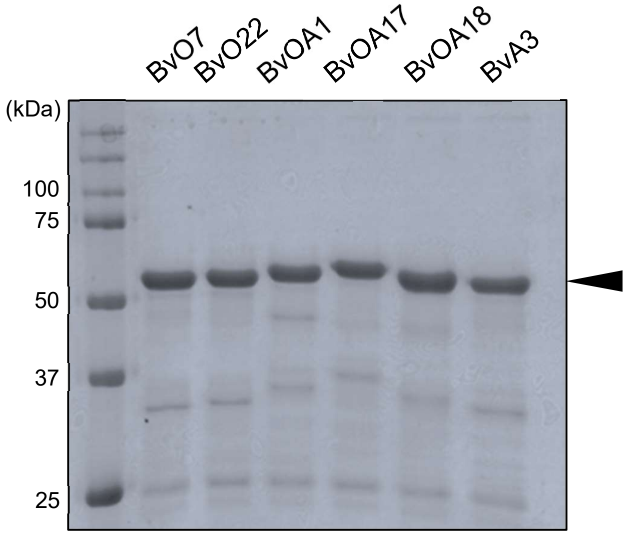

Figure S2. Preliminary SPCE tests to select representative bovine scFv clones. Binding of biotin-labeled, O type-specific (A) and pan-serotype specific (B) scFvs to FMDV type O antigen (O1 Manisa) in the presence of 1/10 dilution of respective serum was detected using NA-HRP. The test serum samples were derived from vaccinated cattle and FMDV (O/Anseong/SKR/2002)-challenged pigs. One hundred percent binding corresponds to the value of absorbance obtained in the presence of FBS.

A

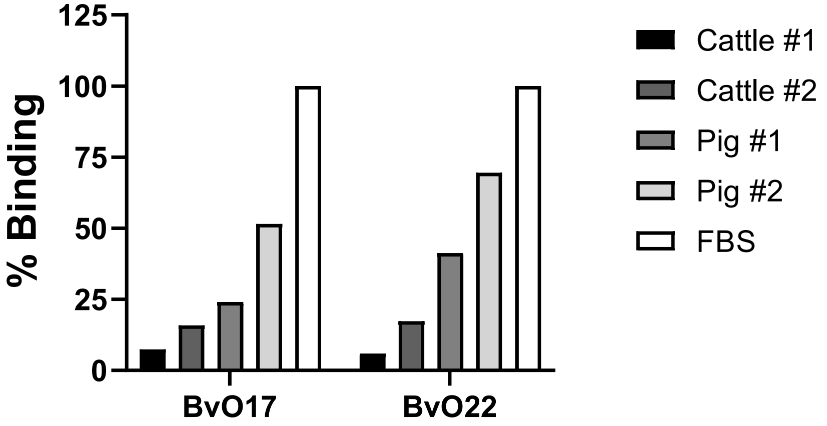

B

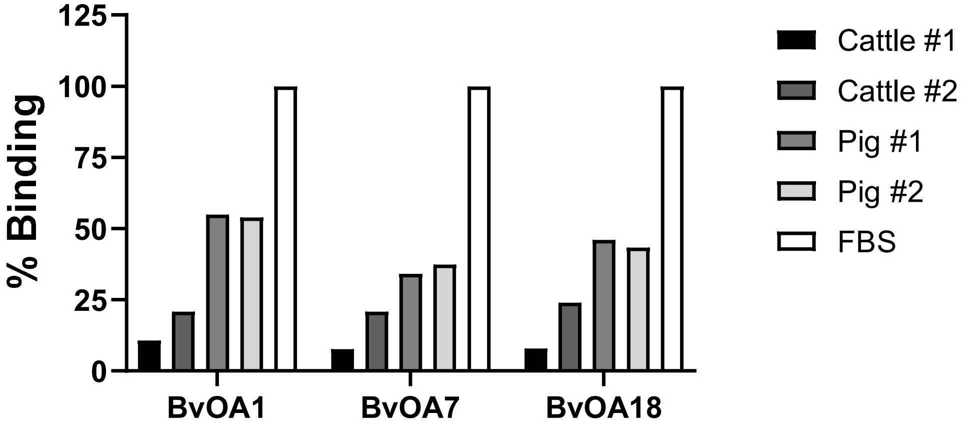

Figure S3. Bovine scFv antibody-based SPCE using positive control anti-sera for seven serotypes of FMDV. (A, D) PrioCHECK FMDV type O and A antibody ELISA kits were used for comparison. The control sera purchased from Pirbright Insti-tute were diluted and tested following manufacturer's instruction. (B, C) Binding of biotin-labeled BvO17 or BvOA7 to FMDV type O antigen (O1 Manisa) in the presence of 1/10 dilution of respective control serum was detected using NA-HRP. (E, F) Binding of BvA3 or biotin-labeled BvOA7 to FMDV type A antigen (A22 Iraq) in the presence of 1/10 dilution of respective control serum was detected using HRP-conjugated anti-human IgG or NA-HRP, respectively. For all the SPCE experiments, the serum samples were tested in triplicate. One hundred percent binding corresponds to the value of absorbance obtained in the presence of negative control serum.

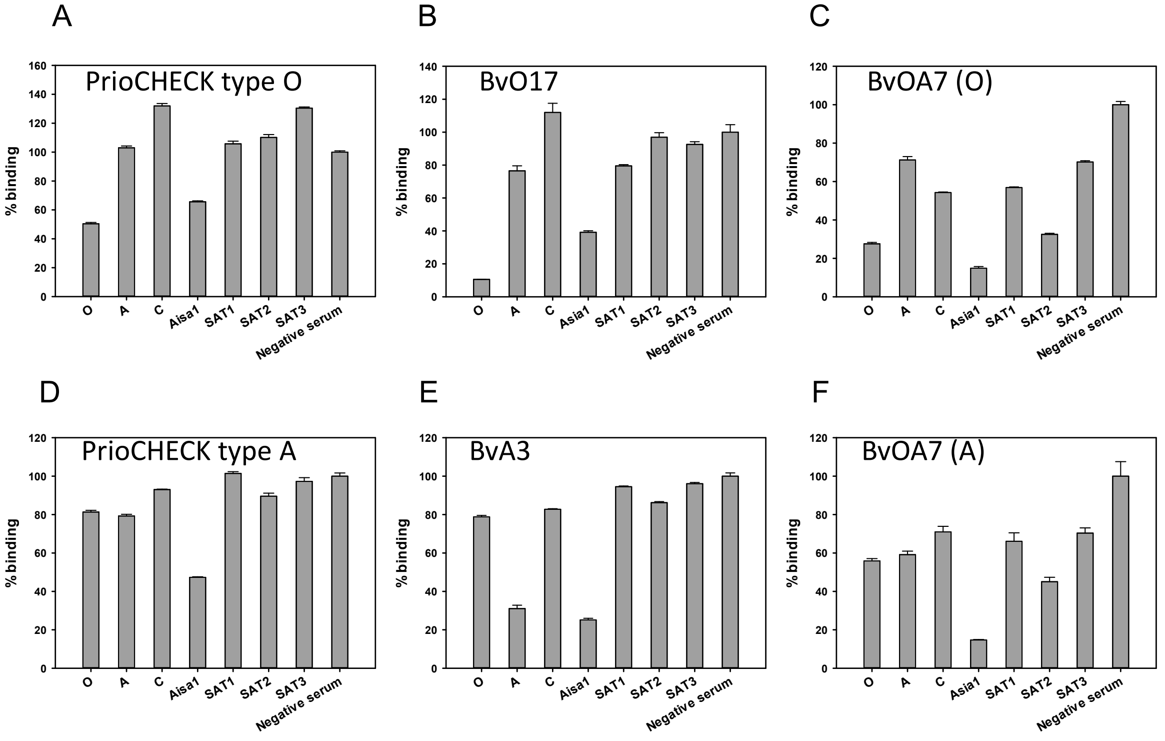

Table S1. Primer sets for bovine antibody library construction.

BVH Forward primer	Sequences (5'-3')
BVH1-2	5’-GCGGCCCAGCCGGCCATGGCCCAGGTGCAGCTGCGGGAGTC-3’
BVH1Q	5’-GCGGCCCAGCCGGCCATGGCCCAGGTGCAGCTGCAGGAGTC-3’
BVH1K	5’-GCGGCCCAGCCGGCCATGGCCAAGGTGCAGCTGCAGGAGTC-3’
**BJH Reverse primer**	**Sequences (5'-3')**
BJH1R	5’-CGAGCCGCCGCCGCCAGATCCACCTCCACCTGAACCTCCTCCACCTGAGGAGACGGTGACCAGG-3’
BJH2R	5’-CGAGCCGCCGCCGCCAGATCCACCTCCACCTGAACCTCCTCCACCTGAGGAGACGGTGACCTCG-3’
BJH6R	5’-CGAGCCGCCGCCGCCAGATCCACCTCCACCTGAACCTCCTCCACCTGAGGAGACGGTGACCCTG-3’
**BVk Forward primer**	**Sequences (5'-3')**
BVk2	5’-GGATCTGGCGGCGGCGGCTCGGATGTTGTGCTGACCCAGAC-3’
BVk4	5’-GGATCTGGCGGCGGCGGCTCGGACATCCAGGTGACCCAGTC-3’
**BJk Reverse primer**	**Sequences (5'-3')**
BJk1R	5’-CTGCTCGAGGCCTCCCGGGCCTTTGATCTCTACCTTGGTTCC-3’
**BVλ Forward primer**	**Sequences (5'-3')**
BVλ1	5’-GGATCTGGCGGCGGCGGCTCGCAGGCTGTGCTGACTCAGC-3’
BVλ1-6	5’-GGATCTGGCGGCGGCGGCTCGCAGGATGTGCTGACTCAGC-3’
BVλ2	5’-GGATCTGGCGGCGGCGGCTCGCAGTCTGGCCTGACTCAGC-3’
BVλ6-14	5’-GGATCTGGCGGCGGCGGCTCGTCTTCTCAGCTGACTCAGC-3’
BVλ6-156	5’-GGATCTGGCGGCGGCGGCTCGTCCTATGAACTGACCCAG-3’
BVλ7-21	5’-GGATCTGGCGGCGGCGGCTCGCAGCCTGTGCTGACTCAGC-3’
BVλ8-40	5’-GGATCTGGCGGCGGCGGCTCGCAGACTGTGATCCAGGAAC-3’
**BJλ Reverse primer**	**Sequences (5'-3')**
BJλ2R	5’-CTGCTCGAGGCCTCCCGGGCCCAGGACGGTCACTCTGGTCC-3’
BJλ3R	5’-CTGCTCGAGGCCTCCCGGGCCCAGGACGGTCAGTGTGGTCC-3’
**VH Forward extension primer scFv assembly**	**Sequences (5'-3')**
scFv-Fex	5’-GACGACGACGACGACGCGGCCCAGCCGGCCATGGCC-3’
**VL Reverse extension primer scFv assembly**	**Sequences (5'-3')**
scFv-Rex	5’-GACGACGACGACGACCTGCTCGAGGCCTCCCGGGCC-3’
